# Acyl-CoA thioesterase 13 (*ACOT13*) attenuates the progression of autosomal dominant polycystic kidney disease *in vitro* via triggering mitochondrial-related cell apoptosis

**DOI:** 10.18632/aging.206054

**Published:** 2024-08-21

**Authors:** Bin Wang, Qi Yang, Lihe Che, Luyao Sun, Na Du

**Affiliations:** 1 Department of Infectious Disease, The First Hospital of Jilin University, Changchun 130021, China; 2 Department of Pathogenic Biology, School of Basic Medicine, Beihua University, Jilin 132013, China

**Keywords:** autosomal dominant polycystic kidney disease, mitochondria, proliferation, apoptosis, ACOT13

## Abstract

Purpose: Autosomal dominant polycystic kidney disease (ADPKD) is the most common cause of end-stage kidney disease. It has been shown that Acyl-CoA thioesterase 13 (*ACOT13*) level was reduced in renal cystic tissues from ADPKD patients. However, the role of *ACOT13* in ADPKD remains largely elusive.

Methods: The data in the GSE7869 dataset were acquired from the GEO database to determine *ACOT13* level between normal renal cortical tissues and renal cystic tissues. Next, the potential functions of *ACOT13* were explored by gene set enrichment analysis (GSEA). Furthermore, *ACOT13* level in ADPKD cells (WT9-12) was verified by RT-qPCR. The effects of *ACOT13* on WT9-12 cell growth were evaluated using the EdU staining and flow cytometry assays.

Results: Compared to normal group, *ACOT13* mRNA level was obviously reduced in renal cystic tissues and WT9-12 cells. Meanwhile, GSEA results showed that compared to the low *ACOT13* expression group, PI3K-Akt and MAPK signaling pathways were inactivated, and PPAR signaling pathway and fatty acid metabolism were activated in high *ACOT13* expression group. Furthermore, overexpression of *ACOT13* notably reduced WT9-12 cell proliferation and triggered cell cycle arrest. Moreover, *ACOT13* overexpression remarkably triggered apoptosis, increased cleaved caspase 3 protein level, reduced ATP production and induced loss of mitochondrial membrane potential in WT9-12 cells, suggesting that *ACOT13* overexpression could trigger mitochondrial-related apoptosis in WT9-12 cells.

Conclusions: Collectively, our results showed that overexpression of *ACOT13* could suppress WT9-12 cell proliferation and trigger mitochondrial-mediated cell apoptosis, suggesting that *ACOT13* may exert a protective role in ADPKD.

## INTRODUCTION

Autosomal dominant polycystic kidney disease (ADPKD) is a hereditary kidney disease that is caused by mutation in polycystic kidney disease-1 (PKD1) gene or polycystic kidney disease-2 (PKD2) gene [1, 2]. Meanwhile, ADPKD is also a primary cause of end-stage renal disease (ESRD) [3]. ADPKD is characterized by the slow progressive cyst growth within two kidneys, resulting in destruction of the kidney parenchyma and impaired kidney function [4]. Tolvaptan, a Food and Drug Administration (FDA)-approved vasopressin receptor antagonist, has been used to treat ADPKD [5]. Tolvaptan effectively suppresses the kidney volume growth and improves kidney function in ADPKD patients [6]. However, tolvaptan can cause some side effects, such as liver injury, chest pain and headaches, limiting its clinical application [7]. Thus, uncovering novel therapeutic targets are important for improving ADPKD treatment.

Mitochondria, serving as the energy center of the cell, play crucial roles in mediating cell growth and metabolism [8, 9]. They are also involved in maintaining the balance of fluid in kidneys and electrolyte, as well as removing toxins from the blood in kidneys [10]. Mitochondrial dysfunction in kidneys is linked to the occurrence and progression of multiple kidney diseases, such as cystic kidney disease, tubularinterstitial disease and podocytopathy [11]. Meanwhile, mitochondrial dysfunction is also involved in the pathophysiology of ADPKD [12]. The dysfunctional mitochondria and aerobic glycolysis can be observed in cysts in ADPKD kidneys [12]. Recently, researchers have shown that enhancing mitochondrial function could alleviate kidney injury and improve kidney function in kidney diseases [13, 14]. Thus, mitochondria-targeted therapeutics may offer a promising approach to prevent the progression of kidney diseases (e.g. ADPKD) via maintaining mitochondria homeostasis [11, 15].

Acyl-CoA thioesterase 13 (*ACOT13*), also known as THEM2, is a mitochondria-associated acyl-CoA thioesterase (Acot) gene [16]. Our previous study found that *ACOT13* level was obviously reduced in ADPKD patients [17]. However, the role of *ACOT13* in ADPKD remains largely unstudied. In the current research, we found that *ACOT13* overexpression could suppress WT9-12 cell proliferation and triggered mitochondrial-mediated cell apoptosis, suggesting that *ACOT13* may play a protective role in ADPKD.

## MATERIALS AND METHODS

### Data collection

The gene expression profiles of the GSE7869 dataset [including 3 normal renal cortical tissues (normal group) and 18 renal cystic tissues (ADPKD group, including 5 minimally cystic tissue, 5 small cysts, 5 medium cysts, 3 large cysts), processing on the Affymetrix Human Genome U133 Plus 2.0 Array] was acquired from the Gene Expression Omnibus (GEO, https://www.ncbi.nlm.nih.gov/geo/) database.

### Screening of differential expressed genes (DEGs)

The limma R package (version 4.1.0) was applied for evaluating *ACOT13* level between the control and ADPKD groups [18]. According to the median value of *ACOT13* level, samples in the GSE7869 dataset were grouped into high *ACOT13* level (H-*ACOT13*) and low *ACOT13* level (L-*ACOT13*) groups. Next, DEGs between H- and L-*ACOT13* groups were screened using the limma R package. |log2 FC| > 1 and p-value < 0.05 were set as the screening criteria.

### Functional analyses

Gene Ontology (GO) and Kyoto Encyclopedia of Genes and Genomes (KEGG) enrichment analyses were performed on DEGs. GO analysis [including molecular function (MF), biological pathways (BP), and cellular components (CC)] and KEGG analysis were conducted with “clusterProfiler” (version 4.1.0) R package [19]. Moreover, gene set enrichment analysis (GSEA) was also conducted using the “clusterProfiler” R package. |NES|>1 and p-value < 0.05 were considered as the significantly enriched pathways.

### Cell culture and transfection

HK-2 (CL-0109, Procell) and ADPKD cell line WT9-12 (CRL-2833, ATCC) cells were cultured in DMEM (PM150210, Procell) supplemented with 10% FBS (164210, Procell) and 100 units/mL of penicillin and streptomycin (PB180120, Procell) in 5% CO_2_ atmosphere at 37° C.

Lentivirus containing *ACOT13* overexpressing plasmids (*ACOT13* OE) and negative control plasmids (Control) were obtained from Tsingke Biotechnology Co. Ltd. (Beijing, China) WT9-12 cells were added with the lentivirus and 6 μg/ml polybrene and incubated for 24 h at 37° C. After removing the media containing the lentivirus, cells were then cultured in fresh DMEM containing 10% FBS for indicated times.

### RT-qPCR assay

Total RNA was extracted from cells using the Trizol reagent (9108, Takara) and then retrotranscribed to cDNA using the PrimeScript™ RT reagent Kit with gDNA Eraser (RR047A, Takara). Next, amplification was done using the SYBR® Premix Ex Taq kit (RR820A, Takara) on a fluorescence quantitative PCR instrument (ABI-7500, Applied Biosystems). *ACOT13*: forward, 5’-TCTGCTATGCACGGAAAGGG-3’, reverse: 5’-TTTCCTGTGGCCTTGTTGGT-3’; *GAPDH*: forward, 5’-GAGTCAACGGATTTGGTCGT-3’, reverse, 5’-TTGATTTTGGAGGGATCTCG-3’. The relative RNA level was calculated using the 2^-ΔΔCt^ method, and *GAPDH* was used as an internal control.

### Western blot assay

The protein concentration was determined using a BCA protein detection kit (P0010, Beyotime). Next, equal amounts of protein were electrophoretically separated on 10% SDS-PAGE and then loaded onto PVDF membranes. After that, the membranes were probed with anti-ACOT13 (1:1000, ab228011, Abcam), anti-cleaved caspase 3 (1:1000, ab32042, Abcam), anti-Ki67 (1:1000, ab16667, Abcam), anti-PCNA (1:1000, ab92552, Abcam) and anti-β-actin (1:1000, ab8226, Abcam) primary antibodies overnight at 4° C, and then incubated with an HRP-conjugated secondary antibody. The immune blots were visualized by an ECL detection reagent (180-5001, Tanon). The gray value of the blots was analyzed by the Image J software.

### Cell counting kit-8 (CCK-8) assay

WT9-12 cells were cultured in a 96-well plate overnight at 37° C. After incubation for indicated times, CCK-8 reagent (C0037, Beyotime) was added for a further 2 h at 4° C. Next, a microplate reader (Multiskan, Thermo Fisher Scientific, USA) was used for reading the absorbance value at 450 nm.

### EdU staining assay

An EdU cell proliferation detection assay kit (CA1170, Solarbio) was applied for assessing cell proliferation. WT9-12 cells were loaded onto a 24-well plate overnight at 37° C, and then stained with 50 μM EdU for 2 h. After fixing with 4% paraformaldehyde (AR1069, Boster) for 30 min at room temperature, cells were stained with the Apollo solution. Cell nucleus was then stained with Hoechst 33342 reagent for 30 min in darkness. Finally, a fluorescence microscope (CKX31, Olympus) was used to observe the EdU positive cells. Meanwhile, the results were analyzed by the Image J software.

### Flow cytometry assay

For cell cycle detection, WT9-12 cells were fixed with 75% pre-cooled ethanol overnight at 4° C. Next, cells were treated with RNAase A (20 μg/ml, EN0531, Thermo Fisher Scientific) for 30 min, and then stained with PI reagent (50 μg/ml, R37169, Invitrogen) for 30 min in darkness. After that, cell cycle distribution was analyzed by a flow cytometer (FACSVerse, BD, USA).

For cell apoptosis detection, an Annexin V-FITC/PI cell apoptosis detection kit (40302ES20, Yeasen) was used. WT9-12 cells were suspended in Annexin-binding buffer, and then stained with FITC-Annexin V (5 μl) and PI (10 μl) reagents for 15 min in darkness. After that, the apoptotic cells were tested by a flow cytometer (FACSVerse, BD, USA).

### Measurement of ATP levels

According to the manufacturer’s protocols, ATP levels in WT9-12 cells were measured by the ATP Detection kit (S0026, Beyotime). Briefly, cells were lysed using the lysis buffer, and then centrifuged at 12000 *g* at 4° C for 5 min. The cell supernatant was collected. 100 μL of ATP reagent was added into a tube for 5 min at room temperature. Next, 20 μL of supernatant were then added into each tube. The results were detected by using a luminometer (Thermo Fisher Scientific, USA).

### Detection of mitochondrial membrane potential (MMP)

The MMP of WT9-12 cells was evaluated by a MMP detection kit with JC-1 (M8650, Solarbio). WT9-12 cells were cultured in a 6-well plate overnight at 37° C, and then stained with JC-1 staining solution at 37° C for 20 min. A fluorescence microscope (CKX31, Olympus) was applied to capture the fluorescence signals of the JC-1 aggregates (red color) and monomers (green color). The MMP level of cells was considered as the ratio of red/green fluorescence intensity.

### Statistical analysis

Each experiment was independently repeated at least three times. Data were expressed as mean ± standard deviation (SD). The differences between two groups were detected with an unpaired Student t-test. P < 0.05 indicates statistical significance.

### Availability of data and materials

The dataset GSE7869 analyzed in this study was acquired from the Gene Expression Omnibus (GEO) database (https://www.ncbi.nlm.nih.gov/geo/).

## RESULTS

### ACOT13 was reduced in renal cystic tissues

**Figure 1:**
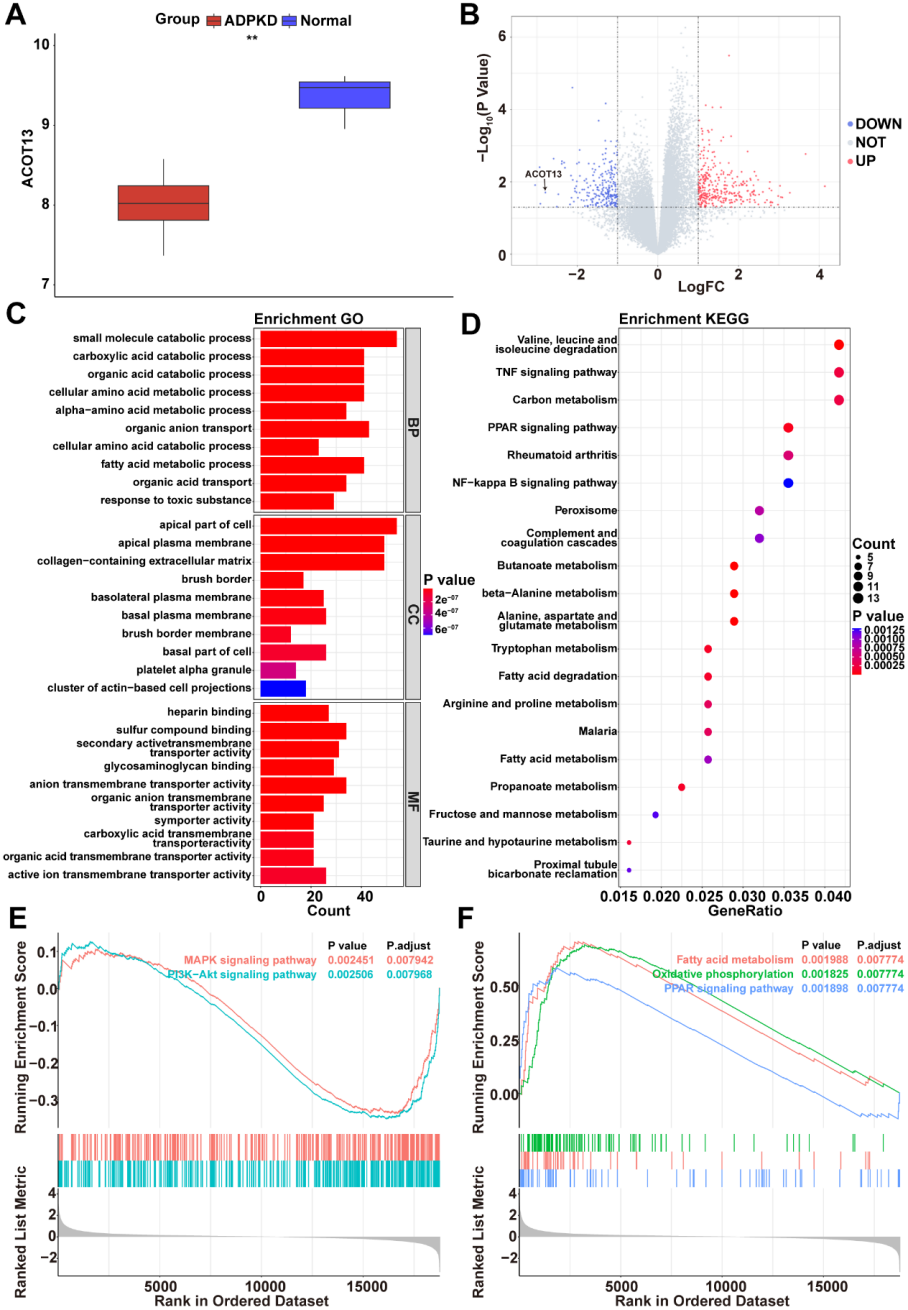
***ACOT13* was reduced in renal cystic tissues.** (**A**) The box plot showed the *ACOT13* level in normal and ADPKD tissues in the GSE7869 dataset. (**B**) The volcano plot displayed DEGs between H- and L-*ACOT13* groups. Blue color represents downregulated genes; red color indicates upregulated genes. (**C**) GO analysis including top 10 BP, top 10 CC and top 10 MF terms. (**D**) Top 20 KEGG pathways. (**E**) Enrichment of genes in the MAPK signaling pathway and PI3K-AKT signaling pathway by GSEA. (**F**) Enrichment of genes in the fatty acid metabolism, oxidative phosphorylation and PPAR signaling pathway by GSEA.

Based on the data in the GSE7869 dataset, we found that compared to normal renal cortical tissues, *ACOT13* level was obviously reduced in renal cystic tissues (Figure 1A). Additionally, the GSE7869 dataset included four types of renal cystic tissues (minimally cystic tissue, small cysts, medium cysts, large cysts). As shown in Supplementary Figure 1, *ACOT13* level was notably reduced in these four types of renal cystic tissues compared to normal renal cortical tissues. Among these, *ACOT13* level was the lowest in small cysts (Supplementary Figure 1).

To analyze the possible pathways associated with *ACOT13* in ADPKD, DEGs were screened between H- and L-*ACOT13* groups. Compared to the L-*ACOT13* group, a total of 632 DEGs were found in the H-*ACOT13* group (Figure 1B). Next, different functional analyses were performed on these 632 DEGs. GO results showed that these genes were appeared in 1398 GO terms (e.g. small molecule catabolic process, apical part of cell and heparin binding) (Figure 1C and Supplementary Table 1). KEGG results showed that 58 KEGG pathways (e.g. PPAR signaling pathway, NF-kappa B signaling pathway, FoxO signaling pathway) were related to these genes (Figure 1D and Supplementary Table 1). Moreover, GSEA results showed that compared to the L-*ACOT13* group, 177 pathways including PI3K-Akt signaling pathway and MAPK signaling pathway (two mitochondrial dependent apoptosis pathways) [20, 21], and PPAR signaling pathway (a pathway that could regulate mitochondrial function) [22], and fatty acid metabolism and oxidative phosphorylation (two processes that occurs in mitochondria) [23], were more enriched in the H-*ACOT13* group (Figure 1E, 1F and Supplementary Table 2). The data in Figure 1E, 1F showed that compared to the L-*ACOT13* group, PI3K-Akt and MAPK signaling pathways were inactivated in the H-*ACOT13* group, and PPAR signaling pathway, fatty acid metabolism and oxidative phosphorylation were activated in the H-*ACOT13* group. These results showed that *ACOT13* may play a role in regulating mitochondrial function in ADPKD.

### The relationship between ACOT13 and mitochondria-associated genes (Mito-RGs) in ADPKD

**Figure 2:**
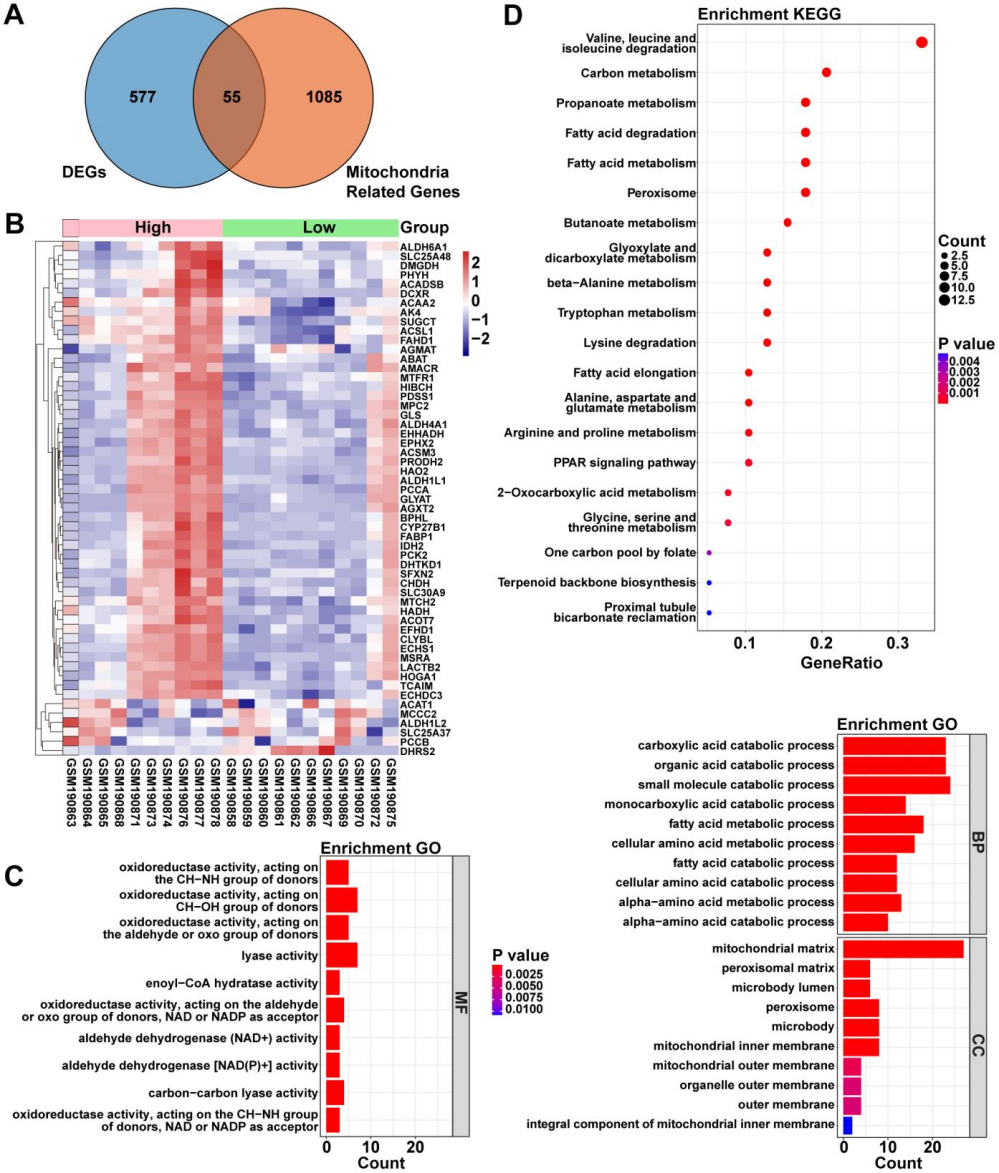
**The relationship between *ACOT13* and mitochondria-associated genes (Mito-RGs) in ADPKD.** (**A**) The common genes (mitochondria-related DEGs) between DEGs and mitochondria-associated genes were screened by Venn. (**B**) Heatmap showed the mitochondria-related DEGs between H- and L-*ACOT13* groups. Blue color represents downregulated genes; red color indicates upregulated genes. (**C**) GO analysis including top 10 BP, top 10 CC and top 10 MF terms. (**D**) Top 20 KEGG pathways.

1140 Mito-RGs were acquired from the MitoCarta3.0 database (http://www.broadinstitute.org/mitocarta). To explore the relationship between *ACOT13* and Mito-RGs in ADPKD, the venn diagram was used to screen the overlapping mitochondria-related DEGs. As shown in Figure 2A, 2B, there were 55 mitochondria-related genes between H- and L-*ACOT13* groups. GO and KEGG analyses were then performed on these 55 genes. GO results showed that these genes were appeared in 329 GO terms (e.g. carboxylic acid catabolic process, mitochondrial matrix, fatty acid oxidation and lipid oxidation) (Figure 2C and Supplementary Table 3). KEGG results showed that 25 KEGG pathways (e.g. Fatty acid metabolism and PPAR signaling pathway) were related to these genes (Figure 2D and Supplementary Table 3).

### ACOT13 was downregulated in an ADPKD cell line (WT9-12)

**Figure 3:**
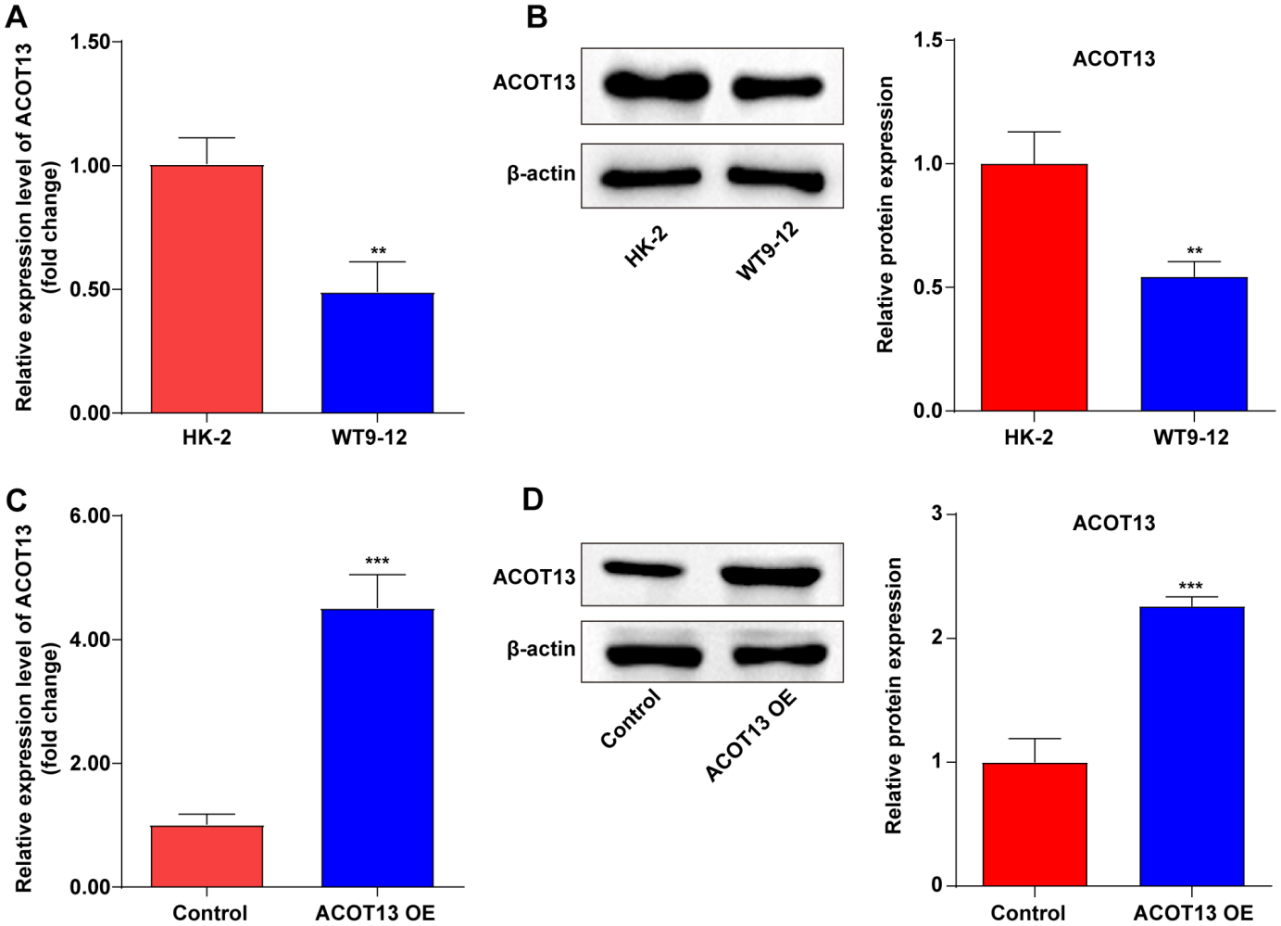
***ACOT13* was downregulated in WT9-12 cells.** (**A**) RT-qPCR was used to determine *ACOT13* mRNA level in HK-2 and WT9-12 cells. **P<0.01 vs. HK-2 group. (**B**) Western blot assays were applied to assess ACOT13 protein level in HK-2 and WT9-12 cells. **P<0.01 vs. HK-2 group. (**C**) RT-qPCR was used to determine *ACOT13* mRNA level in WT9-12 cells transfected with *ACOT13* OE plasmids. ***P<0.001 vs. control group. (**D**) Western blot assays were applied to assess ACOT13 protein level in WT9-12 cells transfected with *ACOT13* OE plasmids. ***P<0.001 vs. control group.

To explore the role of *ACOT13* in ADPKD *in vitro*, *ACOT13* level was evaluated in WT9-12 cells first. The data showed that the mRNA and protein level of ACOT13 was obviously reduced in WT9-12 cells compared to HK-2 cells (Figure 3A, 3B). Meanwhile, compared to the control group, the mRNA and protein level of ACOT13 was remarkably elevated in WT9-12 cells transfected with *ACOT13* OE plasmids (Figure 3C, 3D).

### ACOT13 overexpression suppressed WT9-12 cell viability and proliferation

**Figure 4:**
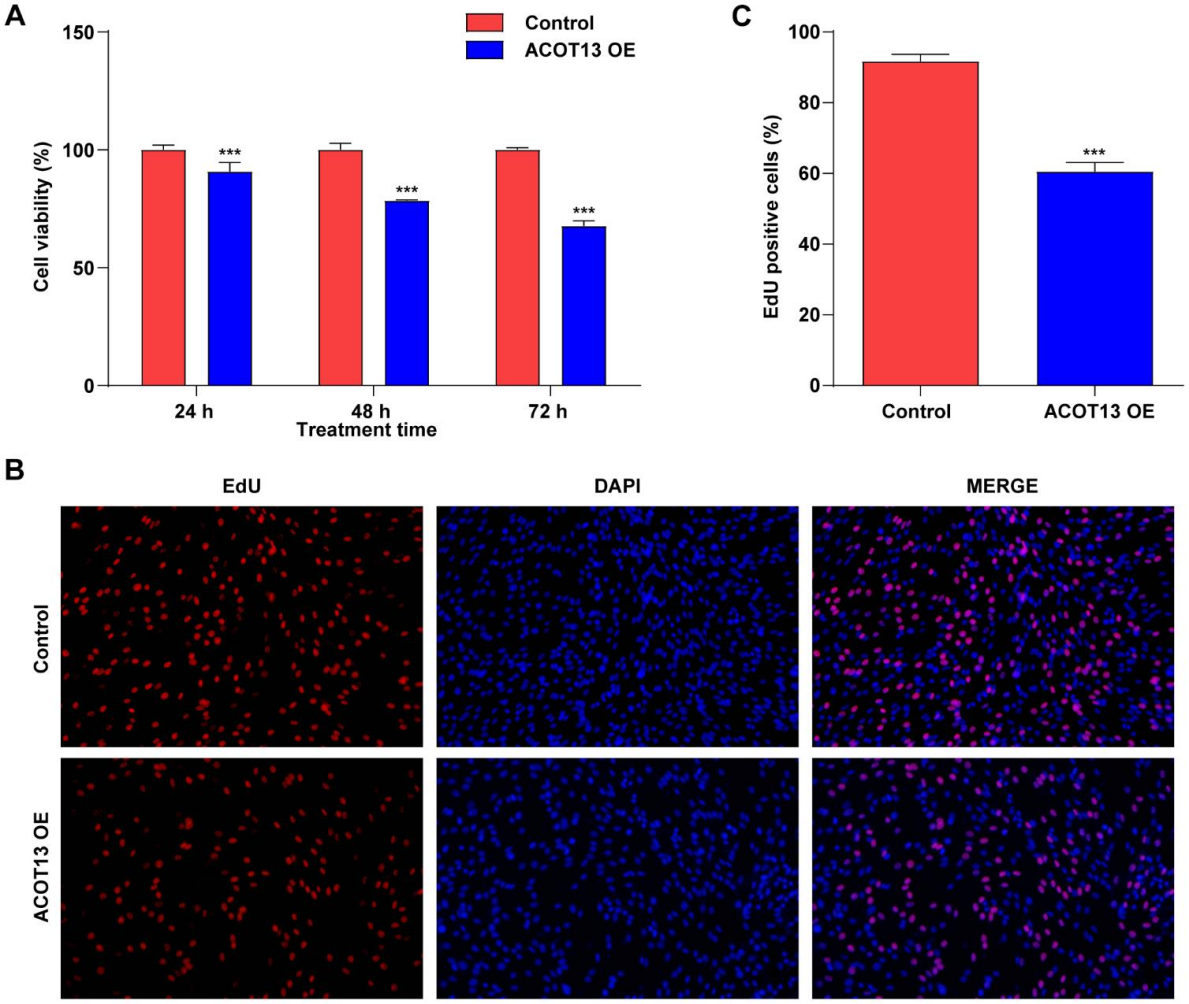
***ACOT13* overexpression suppressed WT9-12 cell viability and proliferation.** WT9-12 cells were transfected with *ACOT13* OE plasmids. Cell viability and proliferation were determined using the (**A**) CCK-8 and (**B**, **C**) EdU staining (magnification, 200×) assays, respectively. ***P<0.001 vs. control group.

Next, the functional roles of *ACOT13* in WT9-12 cells were then investigated by CCK-8 and EdU staining assays. *ACOT13* overexpression induced about 9% growth inhibition in WT9-12 cells at 24 h, induced about 22% growth inhibition in WT9-12 cells at 48 h and induced about 32% growth inhibition in WT9-12 cells at 72 h (Figure 4A), suggesting that *ACOT13* overexpression could decline WT9-12 cell viability. Additionally, *ACOT13* overexpression obviously decreased the percentage of EdU positive cells (~61%) compared with control group (~92%), indicating that *ACOT13* overexpression could suppress WT9-12 cell proliferation (Figure 4B, 4C). Collectively, *ACOT13* overexpression was able to suppress WT9-12 cell viability and proliferation.

### ACOT13 overexpression triggered WT9-12 cell cycle arrest and apoptosis

**Figure 5:**
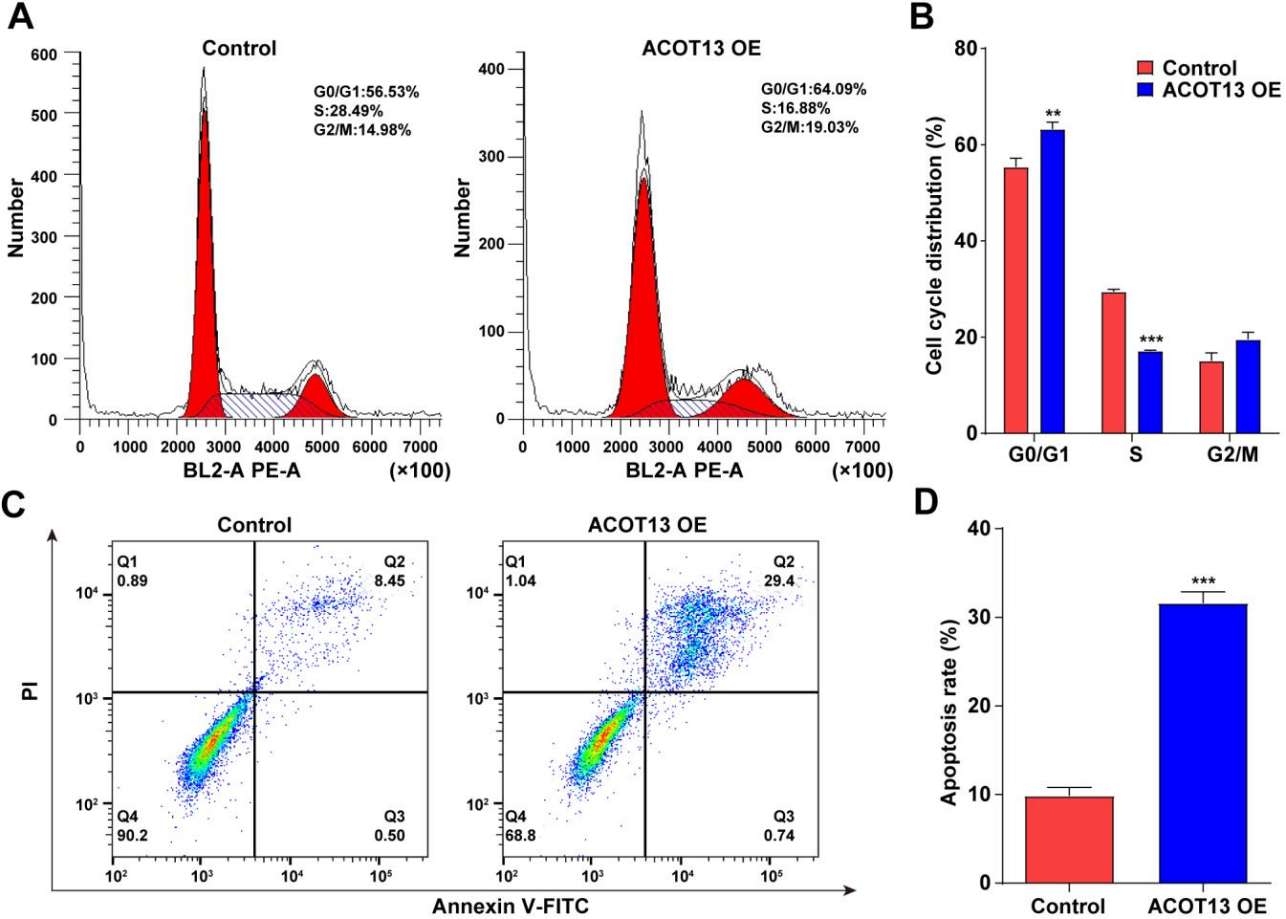
***ACOT13* overexpression triggered WT9-12 cell cycle arrest and apoptosis.** WT9-12 cells were transfected with *ACOT13* OE plasmids. (**A**, **B**) Cell cycle distribution and (**C**, **D**) cell apoptosis were analyzed by the flow cytometry analysis. **P<0.01, ***P<0.001 vs. control group.

We further explored the effects of *ACOT13* on the cell cycle and apoptosis in WT9-12 cells. As revealed in Figure 5A, 5B, compared with control group (~55%), the percentage of WT9-12 cells in the G0/G1 phase increased to ~ 63% after transfection with *ACOT13* OE. Meanwhile, the percentage of *ACOT13* OE-transfected WT9-12 cells in the S phase declined to ~ 17% compared with control group (~29%) (Figure 5A, 5B). Collectively, *ACOT13* overexpression could lead to an increase in the G0/G1 phase population, but a reduction in the S phase population in WT9-12 cells, suggesting that *ACOT13* overexpression could induce cell cycle arrest at G0/G1 phase. Additionally, *ACOT13* overexpression obviously elevated the percentage of apoptotic cells (~32%) compared with control group (~10%), indicating that *ACOT13* could trigger WT9-12 cell apoptosis (Figure 5C, 5D). To sum up, *ACOT13* could induce WT9-12 cell cycle arrest and apoptosis.

### ACOT13 overexpression induced mitochondria damage in WT9-12 cells

**Figure 6:**
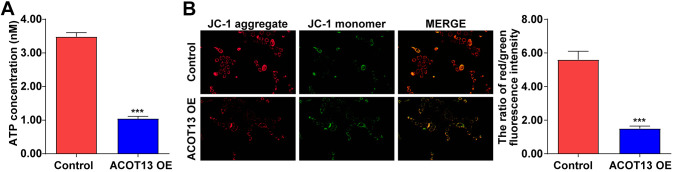
***ACOT13* overexpression induced mitochondria damage in WT9-12 cells.** WT9-12 cells were transfected with *ACOT13* OE plasmids. (**A**) The ATP concentration in cells were detected using an ELISA kit. (**B**) JC-1 staining was performed to evaluate the MMP level (the ratio of red/green fluorescence intensity) in WT9-12 cells (magnification, 200×). ***P<0.001 vs. control group.

Mitochondria could provide cellular energy for cell growth through the generation of ATP [24, 25]. When mitochondria are damaged, a decreased ATP production and a loss of MMP were observed in damaged cells [26]. Thus, we explored the effect of *ACOT13* on mitochondria function in WT9-12 cells. As indicated in Figure 6A, compared with control group, the ATP level was reduced by 70% in *ACOT13* OE-transfected WT9-12 cells, indicating that overexpression of *ACOT13* could decline the ATP level in WT9-12 cells. Additionally, upregulation of *ACOT13* obviously reduced the MMP level in WT9-12 cells compared to control group (Figure 6B). These results illustrated that forced expression of *ACOT13* could induce mitochondria damage in WT9-12 cells.

### ACOT13 overexpression affected proliferation- and apoptosis-related proteins in WT9-12 cells

**Figure 7:**
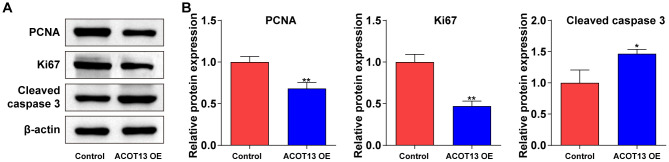
***ACOT13* overexpression affected proliferation- and apoptosis-related proteins in WT9-12 cells.** WT9-12 cells were transfected with *ACOT13* OE plasmids. (**A**, **B**) Western blot assay was used to measure PCNA, Ki67 and cleaved caspase 3 expression levels in WT9-12 cells. *P<0.05, **P<0.01 vs. control group.

Next, Western blot was performed to evaluate proliferation- (PCNA and ki67) and apoptosis-related (cleaved caspase 3) proteins in WT9-12 cells. As shown in Figure 7A, 7B, overexpression of *ACOT13* notably declined PCNA and ki67 levels and increased cleaved caspase 3 levels in WT9-12 cells compared with control group. These results showed that *ACOT13* overexpression could affect WT9-12 cell proliferation and apoptosis via modulating PCNA, ki67 and cleaved caspase 3 levels.

## DISCUSSION

It has been shown that many fluid-filled cysts can be observed in the kidneys of ADPKD patients [27]. With the cyst size increase, cysts can cause stress on normal kidney tissues and may lead to a gradual decline in kidney function, eventually leading to ESRD [27]. Promoting the enlargement of renal cysts could accelerate ADPKD progression [28]. Conversely, inhibiting cyst enlargement and proliferation could prevent the development of ADPKD [29]. For example, Shen et al. found that sorting nexin 9 (*SNX9*) level was notably reduced in ADPKD patients, and *SNX9* overexpression was able to suppress WT9-12 cell proliferation and prevent renal cyst formation [30].

Elliott et al. indicated that the phosphorylation level of c-Src was strongly elevated in ADPKD epithelial cells, inactivation of c-Src could suppress ADPKD cell proliferation and extracellular matrix-attachment *in vitro*, as well as reduce cyst formation in ADPKD mice *in vivo* [31]. Li et al. reported that *DJ-1* upregulation was capable of improving renal dysfunction and reducing cyst growth in kidney tissues of ADPKD mice [32]. Xu et al. showed that inhibition of *COX-2* was able to suppress ADPKD epithelial cell proliferation and trigger cell apoptosis [33]. These findings demonstrated that a number of genes were dysexpressed in ADPKD, and play important roles in ADPKD development. In the current research, we confirmed that the mRNA and protein level of ACOT13 were greatly declined in ADPKD tissues as well as in ADPKD cell line (WT9-12). *ACOT13* overexpression could notably suppress WT9-12 cell proliferation and cell cycle progression and trigger cell apoptosis. Consistent with the previous studies, we found that *ACOT13* could attenuate ADPKD progression via inhibiting cell proliferation and inducing cell apoptosis [30, 33]. For the first time, we demonstrated that *ACOT13* plays important roles in ADPKD development.

*ACOT13* (*Them2*) is a mitochondria-associated Acot gene, which could regulate fatty acids, lipid and glucose metabolism [34–36]. It has been shown that mitochondrial dysfunction and dysregulation of cell metabolism is closely related to the pathogenesis of ADPKD [37, 38]. Fatty acid metabolism is a kind of energy metabolism including fatty acid oxidation and synthesis, which plays crucial roles in maintaining normal physiological function of the body [39]. Fatty acid β-oxidation occurs primarily in mitochondria and it has been found to be impaired in ADPKD [23, 40]. Regaining fatty acid β-oxidation may be a promising approach to attenuate renal injury in mice with chronic kidney disease [41]. Kawano et al. indicated that *ACOT13* could enhance mitochondrial oxidation of fatty acids in liver [35], revealing an association between *ACOT13* and fatty acid oxidation in mitochondria. Meanwhile, in this study, we found that *ACOT13* overexpression could reduce WT9-12 cell proliferation, demonstrating that *ACOT13* exerted a protective role in ADPKD. Additionally, to further explore the functional role of *ACOT13* in ADPKD, DEGs were screened between H- and L-*ACOT13* groups in the GSE7869 dataset and these DEGs may be participated in the protective role of *ACOT13* in ADPKD. Thereafter, to uncover potential signaling pathways associated with these DEGs, GSEA was conducted. The results showed that compared to the L-*ACOT13* group, PPAR signaling pathway and fatty acid metabolism were more activated in the H-*ACOT13* group. Thus, we suspected that *ACOT13* may attenuate ADPKD progression via affecting fatty acid metabolism. This is the first time to show the relationship between *ACOT13* and fatty acid metabolism in ADPKD. Additionally, PPARs are fatty acid-activated transcription factors playing a crucial role in maintaining energy metabolism (e.g. fatty acid β-oxidation) [42, 43]. Lakhia et al. reported that PPARα overexpression could inhibit kidney cyst proliferation in ADPKD through enhancing fatty acid β-oxidation [23]. Collectively, we suspected that *ACOT13* may regulate mitochondrial fatty acid β-oxidation through PPAR signaling, thereby attenuating ADPKD progression. Nevertheless, further studies are needed to clarify the molecular mechanism of *ACOT13* in ADPKD in the future.

In ADPKD, the abnormal increase in renal epithelial cell growth leads to the development of numerous fluid-filled cysts, and a progressive renal dysfunction [44]. Ki67 and PCNA are commonly used as markers for cell proliferation, and high levels of ki67 and PCNA are associated with increased cell growth [45, 46]. In this study, we found that *ACOT13* overexpression could decline PCNA and ki67 protein levels WT9-12 cells, suggesting that *ACOT13* overexpression could inhibit WT9-12 cell proliferation through downregulating PCNA and ki67.

Cell apoptosis exerts key roles in ADPKD development [47]. Evidence have shown that apoptosis is an early event in ADPKD [48, 49]. For example, Lin et al. found that apoptosis exerted an essential role in the formation of Madin-Darby canine kidney cell cysts, and inhibition of cell apoptosis could suppress the cystogenesis [50]. Conversely, worsening ADPKD may related to the declined apoptosis [47]. Chen et al. showed that MIF knockdown notably triggered cyst-lining epithelia cell apoptosis, and suppressed renal cyst formation in ADPKD mice [51]. Consistent with Chen et al.’s finding, our results showed that *ACOT13* overexpression obviously triggered WT9-12 cell apoptosis via upregulating cleaved caspase 3, suggested that *ACOT13* could attenuate ADPKD progression via inducing cell apoptosis. Furthermore, *ACOT13* overexpression strongly reduced ATP production and MMP level in WT9-12 cell, suggesting that *ACOT13* could lead to mitochondria damage in WT9-12 cells. Mitochondria-related apoptosis has been reported to be related to the loss of MMP and reduced ATP generation [52]. These data demonstrated that *ACOT13* could induce mitochondrial-mediated apoptosis in WT9-12 cells. Additionally, GSEA results showed that PI3K/Akt and MAPK signaling pathways were obviously inactivated in the H-*ACOT13* group. PI3K/Akt and MAPK signalings are two mitochondrial dependent apoptosis pathways [53]. Inhibition of PI3K/Akt and MAPK/ERK signalings could lead to mitochondrial-mediated cell apoptosis [54]. Therefore, we suspected that *ACOT13* may enhance mitochondrial-mediated cell apoptosis via inactivating PI3K/Akt and MAPK signalings, which needed to be further explored in the future.

## CONCLUSIONS

Collectively, our results showed that *ACOT13* level was greatly reduced in WT9-12 cells as well as in renal cystic tissues. Overexpression of *ACOT13* remarkably suppressed WT9-12 cell proliferation and triggered mitochondrial-mediated cell apoptosis, suggesting that *ACOT13* may exert a protective role in ADPKD. These findings revealed that *ACOT13* may be a potential option for the treatment of ADPKD.

## Supplementary Material

Supplementary Figure 1

Supplementary Table 1

Supplementary Table 2

Supplementary Table 3
