# Supplementary material for: Acyl-CoA thioesterase 13 (ACOT13) attenuates the progression of autosomal dominant polycystic kidney disease in vitro via triggering mitochondrial-related cell apoptosis
Source: Aging (Albany NY). 2024 Aug 21;16(16):11877–92. doi: 10.18632/aging.206054 (PMC11386924; doi:10.18632/aging.206054)
Supplement: Supplementary Figure 1 [file aging-16-206054-s001.pdf]

SUPPLEMENTARY FIGURE

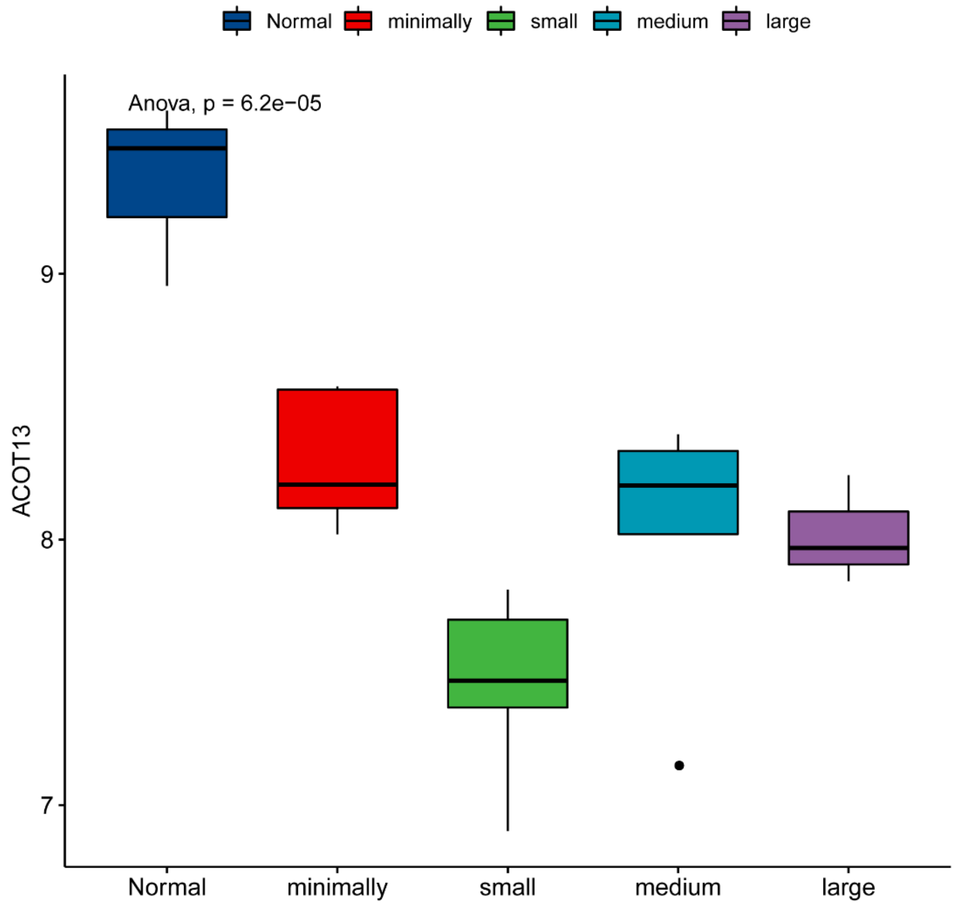

Supplementary Figure 1. The mRNA level of *ACOT13* in different cysts. The box plot showed the *ACOT13* level in normal renal cortical tissue, minimally cystic tissue, small cysts, medium cysts, large cysts in the GSE7869 dataset.
